# Dentition‐Cognition Relationship in Aging Populations: A Meta‐Analysis of Longitudinal Data

**DOI:** 10.1111/joor.70149

**Published:** 2026-01-19

**Authors:** Huimin Chen, Ling Ji, Yilin Wang, Iven Klineberg, Hui Chen

**Affiliations:** ^1^ Division of Restorative Dental Sciences, Faculty of Dentistry The University of Hong Kong Hong Kong SAR P.R. China; ^2^ Second Affiliated Hospital, Chinese University of Hong Kong, Shenzhen & Longgang District People's Hospital of Shenzhen Shenzhen China; ^3^ School of Dentistry, Faculty of Medicine and Health The University of Sydney Sydney New South Wales Australia; ^4^ Oral Restorative Sciences Westmead Centre for Oral Health Westmead New South Wales Australia

**Keywords:** dementia, longitudinal study, meta‐analysis, mild cognitive impairment, tooth loss

## Abstract

**Background:**

Recent research suggests a potential link between tooth loss and cognitive decline among the elderly population, but longitudinal evidence remains limited.

**Aim:**

This meta‐analysis aims to investigate the longitudinal relationship between dentition status (tooth loss/edentulism) and cognitive outcomes (dementia/MCI) in aging populations.

**Method:**

A systematic search was conducted across PubMed, Scopus, and Web of Science up to February 2025. Twenty‐one longitudinal studies (*N* = 35 744 989 participants) meeting inclusion criteria were analysed. Pooled odds ratios (ORs) with 95% confidence intervals (CIs) were calculated using random‐ or fixed‐effects models. Study quality was assessed via the Newcastle‐Ottawa Scale.

**Results:**

Longitudinal evidence supports a connection between tooth loss and cognitive decline. Specifically, tooth loss was associated with increased risks of dementia (OR = 1.26 [1.07, 1.49]) and MCI (OR = 1.40 [1.14, 1.71]). Edentulism showed higher risks (dementia: OR = 1.16 [1.09, 1.23]; MCI: OR = 1.90 [1.07, 3.35]). Subgroup analyses revealed greater risks in women and Western populations. Denture use mitigated dementia risk in individuals with tooth loss (OR = 1.03 [0.82, 1.28]).

**Conclusion:**

Tooth loss may accelerate cognitive decline, with more severe loss correlating to greater deterioration. Dentures could help mitigate this effect by restoring chewing function. Given that tooth loss is a modifiable risk factor for cognitive impairment, preventive dental care and timely prosthodontic treatment may play a protective role in maintaining brain health among older adults.

## Graphical abstract

Tooth loss is a risk factor for cognitive impairment in longitudinal studies. The application of dentures can alleviate the side impact of tooth loss on cognitive decline.

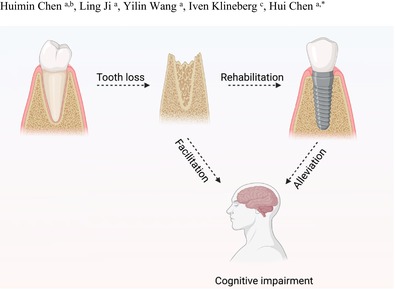

## 1 Introduction

Dementia is a prominent global issue, particularly in aging populations. According to the Global Burden of Disease (GBD) 2019 Dementia Forecasting Contributors, the anticipated rise in the number of individuals diagnosed with dementia will rise from 57.4 million to 152.8 million by 2050 [1]. Dementia is featured by language disability, memory deficit, and mobility dysfunction, with Alzheimer's disease (AD) referred to be the most prevalent subtype [2, 3, 4]. Other than affecting the quality of life of patients, dementia generates considerable economic burdens on the healthcare system, communities, and families [5, 6]. As a progressive and incurable condition, dementia is expected to pose substantial challenges to future societies, especially in the context of increasingly severe aging populations. Mild cognitive impairment (MCI), which is commonly considered as the early middle stage of cognitive decline, can be difficult to distinguish from normal aging due to its tendency to not interfere with autonomous daily activities [7]. Due to the fact that MCI can progress and converse into dementia, it often works as an early indicator of dementia [8].

Since dementia is highly prevalent and devastating, targeting changeable risk factors is critical for prevention. Numerous studies have explored potential risk factors contributing to cognitive impairment. Several common and unmodifiable risk factors have been determined, including gender, race, cardiovascular disease, diabetes, and unhealthy lifestyle habits [7, 9]. In recent 20 years, oral health has become to be identified as a relevant factor in cognitive function and has gained accumulating attention [10, 11].

Tooth loss, a prevalent oral health condition, affects individuals worldwide. According to a report by the World Health Organization (WHO), the prevalence of complete tooth loss is estimated to be 23% among individuals aged 60 and above, with a huge disparity between low‐income countries (11.7%) and high‐income countries (25.4%) [12]. Generally, tooth loss has a negative impact on linguistic function, aesthetics, mastication, self‐esteem, and the well‐being related to oral health [13, 14]. Furthermore, in addition to altering dietary patterns, tooth loss may directly contribute to digestive system disorders, including gastric irritation [15, 16].

Growing research points to a possible relationship among tooth loss and impaired cognitive function. Earlier cross‐sectional research demonstrated that preserving more teeth correlated with better cognitive performance, with a cutting‐off point of 20.5 which is close to the guided number of ‘8020’ recommended by WHO [17, 18]. Tooth loss in the mice model was found to cause spatial learning disability due to the decreased myelin sheath and postsynaptic [19]. Parallel findings were observed in edentulous individuals, who exhibited a decrease in grey matter volume in memory‐ and cognition‐related regions, including the hippocampus, caudate nucleus, and temporal pole [20]. The subsequent compromised cognitive performance can be partially restored by the use of functional prostheses through improved mastication as prosthetic rehabilitation can increase regional blood flow and regional cerebral activity [21, 22].

Although extensive research has examined the association between dentition status and cognition and the biological pathways involved, however, a causal relationship cannot be determined in cross‐sectional studies. Clarifying this relationship holds significant clinical value, as it may yield preventive strategies against cognitive decline initiation and progression. Therefore, this study aims at investigating the potential causal links between dentition status and cognitive performance through longitudinal studies.

## 2 Methods

### 2.1 Search Strategy

This present study was performed under the guideline of PRISMA [23]. We performed a systematic literature search across three databases (Scopus, PubMed, and Web of Science) for English‐language articles published up to February 2025. The search strategy combined the terms (cognitive decline OR Alzheimer's disease OR dementia) AND (tooth loss OR edentulism). Since this meta‐analysis synthesises data from observational studies (rather than interventional trials), PROSPERO does not require registration for this study type.

The systematic search was carried out independently by two investigators (HMC and LJ) to ensure reliability. The third viewer (HC) would join and make discussions till a consensus was achieved when there is any disagreement. The titles were first examined to find the eligible studies. Then, the abstracts and full texts were scanned. Replicates were removed and the articles that did not fit the inclusion criteria were discarded.

### 2.2 Eligibility Criteria

This study integrated longitudinal and cohort studies that examined cognitive performance as the response variable and tooth loss as the predictor. Additionally, cohort case–control studies with a longitudinal design were also incorporated. Exclusion criteria comprised studies involving animal models, systematic reviews, and cross‐sectional designs. Furthermore, studies were excluded if they: (1) lacked full‐text availability, (2) provided insufficient statistical data (RR, OR, HR, or CI), or (3) focused on non‐cognitive mental disorders.

### 2.3 Study Quality Evaluation and Variable Extraction

The Newcastle‐Ottawa Scale (NOS) was implemented to evaluate the integrity of cohort studies [24]. Studies rated with more than 5 stars were deemed to be of high quality and were included in the present study.

Data extraction focused on studies examining tooth loss as the predictive variable and dementia, Alzheimer's disease, or mild cognitive impairment as the response variables. Dentitions with more than 20 teeth were defined as relatively intact dentitions [18].

### 2.4 Data Analysis

A meta‐analysis was performed to examine the association between tooth loss/edentulism (exposure) and MCI/dementia (outcome). Pooled odds ratios (ORs) with 95% confidence intervals (CIs) were computed to quantify this relationship. The original hazard ratio (HR) values sourced from the literature were transformed and combined with odds ratios (OR) values [25]. Moreover, the β values were converted into an odds ratio (OR) value and incorporated into the meta‐analysis in accordance with the methodology outlined by Chinn [26]. A value of OR < 1 suggests a reduced risk of tooth loss in cognitive performance, while a value of OR > 1 suggests an increased risk of missing teeth in cognitive performance. The *I* ^2^ statistic was employed to quantify heterogeneity. For substantial heterogeneity (*I* ^2^ > 50%), a random‐effects model was implemented, while a fixed‐effects model was implemented for lower heterogeneity levels. Meta‐analysis was conducted in Reviewer Manager (version 5.4). Egger's test, combined with funnel plot, was deployed to detect possible publication bias [27] using R version 4.2.2.

## 3 Results

### 3.1 Characteristics of the Included Studies

**FIGURE 1:**
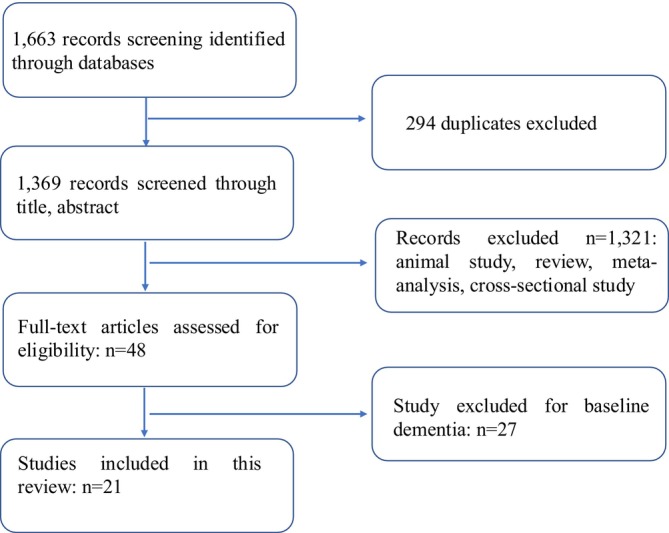
Search process under the PRISMA guideline.

The study selection process is illustrated in Figure 1. Initially, there are 1663 studies scanned in total from three selected databases (Scopus, PubMed, Web of Science). 294 duplicates were removed first. Following initial screening, the titles and abstracts of 1369 potentially relevant articles were evaluated against the inclusion criteria. Following this, 48 full‐length articles were examined, with those focusing on baseline dementia being excluded. Ultimately, 21 eligible studies were analysed quantitatively. The quality assessment was performed using the NOS scale (Table 1), with the included studies receiving scores ranging from 5 to 9.

**TABLE 1:** Assessment of risk of bias using Newcastle‐Ottawa scale.

Longitudinal study	Selection				Comparability	Outcome			
	Representativeness	Selection	Ascertainment	Demonstration	Comparability	Assessment	Follow‐up	Adequacy	Total
Dintica et al. (2018) [28]	+	+	+	+	+	+	+	−	8
He et al. (2023) [29]	+	+	+	+	+	+	+	+	9
Kim et al. (2021) [30]	+	+	+	+	+	+	+	+	9
Ko et al. (2022) [31]	+	+	+	+	−	+	+	+	7
Nagatani et al. (2023) [32]	+	+	+	+	+	+	+	−	8
Okamoto et al. (2015) [33]	+	+	+	+	+	+	+	+	9
Saito et al. (2018) [34]	+	+	+	+	+	+	+	−	8
Xu et al. (2021) [35]	+	+	+	+	+	+	+	−	8
Yamamoto et al. (2012) [36]	+	+	+	+	+	+	+	+	9
Yoo et al. (2019) [37]	+	+	+	+	+	+	+	+	9
Asher et al. (2023) [38]	+	+	+	+	+	+	+	−	9
Chen et al. (2025) [39]	+	+	−	+	−	+	+	+	7
Shimada et al. (2025) [40]	+	+	+	+	+	+	+	+	9
Chou et al. (2024) [41]	+	+	+	+	+	+	+	+	9
Kiuchi et al. (2024) [42]	+	+	−	+	+	+	+	−	7
Miyano et al. (2024) [43]	+	+	−	+	−	+	−	−	5
Yoo et al. (2023) [44]	+	+	+	+	+	+	+	+	9
Kulkarni et al. (2023) [45]	+	+	+	+	+	+	+	+	9
Chen et al. (2024) [46]	+	+	−	+	+	+	+	−	7
Kusama et al. (2023) [47]	+	+	−	+	+	+	+	+	8
Li et al. (2023) [48]	+	+	−	+	+	+	+	+	8

Table 2 provided a summary of the characteristics in the included studies, which were published during the period from 2012 [36] to 2025 [40]. The whole number of participants reached 35 744 989, with a mean tracking period of 8.87 years. Of the 21 included studies, two studies targeted at the Swedish population [28, 29], one study focused on the Finish population [38], one study used data from the TriNetX platform in the US [45], and the other 17 studies focused on the Asian population [30, 31, 32, 33, 34, 35, 36, 37, 39, 40, 41, 42, 43, 44, 46, 47, 48] (8 in Japan, 4 in Korea, and 5 in China).

**TABLE 2:** Description of selected articles.

Authors	Sample size	Follow‐up year	Study population	Cognition tests	Confounding factors	Conclusion
Dintica et al. (2018) [28]	2715	9	Sweden	Professional diagnosis and MMSE (the Diagnostic and Statistical Manual of Mental Disorders, Fourth Edition (DSM–IV) criteria)	Age, gender, level of education, smoking, alcohol intake, presence of multiple health conditions, walking speed, presence of Apolipoprotein ε4 allele, anaemia, low albumin levels, diabetes, C‐reactive protein levels, cardiovascular diseases, and cerebrovascular diseases	*N* = 283 developed into dementia Tooth loss has a significant negative impact on dementia and grey matter volume
He et al. (2023) [29]	14 439	40	Sweden	Professional diagnosis using Swedish versions of the International Classification of Diseases (ICD)	Calendar period in five‐year intervals, gender, residential area, marital status, tobacco use status, alcohol consumption, and CCI	*N* = 1464 developed into dementia Increased risk of tooth loss was related to cognitive decline HR = 1.21 [1.02, 1.42]
Kim et al. (2021) [30]	Control (31 848) AD (7962)	13	Korea	Professional diagnosis (not specified)	Age, gender, income, residential region, obesity, smoking, alcohol intake, systolic blood pressure, diastolic blood pressure, fasting blood glucose, total cholesterol, and CCI	Tooth loss related to cognitive decline for those aged > 60 OR = 1.15 [1.07–1.23] 16 was regarded as the cutting‐off number of tooth loss; No difference in upper and lower tooth loss
Ko et al. (2022) [31]	Control (366) Dementia (122)	9	Korea	MMSE‐K and K‐MMSE	Age and sex	Causative effect was found in tooth number and cognitive decline, OR = 1.195 [1.025–1.393] Alcohol consumption was regarded as a significant confounding factor
Nagatani et al. (2023) [32]	1410	9	Japan	MMSE	Age, gender, years of education, housing type, history of chronic conditions (hypertension, heart disease, stroke, diabetes, cancer), frequency of outdoor activities, and quality of sleep	*N* = 261 had MCI Tooth loss associated with new onset of MCI, HR = 1.30 [1.00–1.69]
Okamoto et al. (2015) [33]	2335	5	Japan	MMI	Age, gender, MMSE (Mini‐Mental State Examination) total score, recall ability, GDS (Geriatric Depression Scale) score, years of education, alcohol consumption, smoking status, history of cancer, myocardial infarction, cerebrovascular disease, diabetes mellitus, hypertension, dyslipidemia, follow‐up details including systolic and diastolic blood pressure, and cerebrovascular disease status	*N* = 241 developed into MCI The progression of tooth loss was associated with MMI, OR = 1.02 [1.00–1.03]
Saito et al. (2018) [34]	140	4	Japan	MMSE	Age, gender, hypertension, diabetes, history of cerebrovascular/cardiovascular disease, hypercholesterolemia, presence of depressive symptoms, body mass index (BMI), current smoking status, current drinking status, years of education, and baseline MMSE (Mini‐Mental State Examination) score	*N* = 241 developed into MCI 0–9 teeth loss is more prone to develop into cognitive decline than other kinds of partial tooth loss, OR = 3.31 [1.07–10.2]
Xu et al. (2021) [35]	11 862	5.93	China	Chinese version of MMSE	Age, gender, enrollment year, province of residence, ethnicity, marital status, occupation, education level, ADL (Activities of Daily Living) score, physical performance score, food diversity score, social activity score, presence of chronic diseases, number of teeth at baseline, and denture wearing status at baseline	*N* = 3966 developed into MCI A higher rate of tooth loss is associated with elevated risk of MCI, HR = 0.94 (0.85–1.03) Denture wearing has a protective effect
Yamamoto et al. (2012) [36]	4425	4	Japan	Professional diagnosis (standardised questionnaire)	Age, gender, income level, health status indicators (BMI, current illness), and health behaviours (smoking history, alcohol consumption, exercise habits)	*N* = 220 developed into dementia Teeth without denture and less dental visit, poorer mastication ability are correlated with cognitive decline, HR = 1.85 [1.04–3.31]
Yoo et al. (2019) [37]	209 806	9	NHIS‐ECD	MMSE, Delayed Word Recall Method, Beck's Depression Inventory, and Verbal Fluency Performance Test	Age, gender, socio‐economic factors (income, residential area, insurance eligibility), and history of dental caries or periodontal disease as potential confounding variables	*N* = 24 044 developed into dementia, OR = 1.18 [1.146–1.215] Women have a bigger odds ratio than men
Asher et al. (2023) [38]	3426	11	Finnish	MMSE	Age, gender, years of education, baseline cognitive scores, living area, cardiovascular risk factors, income level, presence of depression, C‐reactive protein levels, and denture status	Having less than 20 teeth was linked to lower baseline cognition with an odds ratio (OR) of 1.21 (95% CI 1.03–1.43), a 11‐year cognitive decline with an OR of 1.30 (95% CI: 1.05–1.70), and an increased 15‐year dementia risk with a hazard ratio (HR) of 1.52 (95% CI: 1.15–2.02) after adjusting for confounding factors
Chen et al. (2025) [39]	2247	9	Taiwan	Medical diagnosis (not specified)	Age, sex, education level, diabetes status, periodontitis, history of head injury, coronary artery disease (CAD), history of stroke, smoking habits, and alcohol consumption	The presence of fewer than 20 natural teeth or not using dentures was not found to have a significant impact on cognition Having fewer natural teeth without dentures was associated with a significantly higher risk with a hazard ratio (HR) of 1.57 (95% CI: 1.04–2.37)
Shimada et al. (2025) [40]	21 306	7	Japan	MMSE	Age, education level, history of stroke, hypertension, diabetes status, hearing loss, BMI, GDS score, smoking habits, alcohol consumption, marital status, walking time, and psychiatric disorders	Lower income was linked to dementia with a hazard ratio (HR) of 1.18 (95% CI 1.10–1.26), and this association was partially attenuated when controlling for the number of teeth, resulting in an HR of 1.17 (95% CI: 1.09–1.25)
Chou et al. (2024) [41]	96 272	7	Taiwan	SPMSQ	Age, sex, marital status, co‐residence status, education level, income level, smoking habits, alcohol consumption, and BMI	Older adults with greater tooth loss faced an elevated risk of developing cognitive impairment An increase in tooth loss was linked to a higher risk of developing cognitive impairment
Kiuchi et al. (2024) [42]	44 083	10	Japan	Activities of Daily Living Independence Assessment Criteria for Older Individuals with Dementia scale	Age, gender, socio‐economic status, and history of dental caries or periodontal disease	Fewer teeth were associated with dementia, OR = 1.14 (1.07,1.22)
Miyano et al. (2024) [43]	22 687	1.1	Japan	ICD‐10	Age, BMI, smoking, and alcohol consumption, CCI	Partial tooth loss and complete tooth loss are associated with cognitive decline, with odds ratios (OR) of 1.34 (95% CI: 1.01–1.77) and 1.54 (95% CI: 1.03–2.30), respectively
Yoo et al. (2023) [44]	2 555 618	9.2	Korea	ICD‐10	Age, sex, income level, smoking habits, alcohol consumption, physical activity level, BMI, presence of comorbidities, history of dental diseases, and oral hygiene practices	Missing half to one third of the dentition was associated with an increased risk of dementia, with a hazard ratio (HR) of 1.07 (95% CI: 1.02, 1.12). Dental cleaning and teeth brushing can decrease the OR for dementia
Kulkarni et al. (2023) [45]	32 651 565	5	US	ICD‐10	Age, gender, body mass index (BMI), sodium levels, glucose levels, triglycerides, total cholesterol, LDL cholesterol, HDL cholesterol, and C‐reactive protein level	Compared with other oral diseases, tooth loss was the most significant risk factor for Alzheimer's disease, with a relative risk (RR) of 3.186 (95% CI: 3.007, 3.376) Poor oral health increased the risk for cognitive impairment. RR = 2.363 (2.326, 2.401)
Chen et al. (2024) [46]	516	6	Taiwan	MOCA	Age, sex, years of education, APOE e4 status, hypertension, body mass index (BMI), serum levels of *H. pylori* IgG and IL‐6, diabetes, practice effect, and follow‐up period	Impaired dentition was linked to lower scores in attention (^*β* = −0.05) and verbal fluency (^*β* = −0.03), especially among individuals with elevated inflammatory markers
Kusama et al. (2023) [47]	37 556	9	Japan	Three questions related to subjective cognitive complaints (SCCs)	Sex, age, subjective cognitive complaints, income, education level, marital status, smoking status, alcohol consumption, walking time, and comorbidities (cancer, stroke, diabetes, and hypertension)	Having 19 or fewer remaining teeth (HR = 1.12, 95% CI = 1.03–1.23) and being edentulous (having no natural teeth) (HR = 1.20, 95% CI = 1.09–1.32) were significantly associated with a higher risk of dementia
Li et al. (2023) [48]	16 510	5	China	Questionnaire and interview	Age, sex, education, household income, smoking, alcohol use, sleep duration, denture status, BMI, hypertension, diabetes, respiratory illnesses, stroke, heart disease, and other chronic conditions	Individuals with complete tooth loss experienced a more significant decrease in cognitive function compared to those without, with a beta coefficient (β) of −0.70 (95% CI: −1.09, −0.31)

Abbreviations: BMI, body mass index; CCI, Charlson Comorbidity Index; GDS, The global deterioration scale; ICD, international classification of disease; MMSE, Mini‐Mental State Examination; MMSE‐K and K‐MMSE, Korean version of Mini‐Mental State Examination (MMSE‐K) and the Korean MMSE (K‐MMSE); NHIS‐ECD, National Health Insurance Service‐Elderly Cohort Database.

Three studies had a sample population less than 1000 [31, 34, 46], twelve studies had a population of more than 10 000 participants [29, 30, 35, 37, 40, 41, 42, 43, 44, 45, 47, 48], the other six research had a sample population between 1000 to 10 000 [28, 32, 33, 36, 38, 39]. Various methods of dementia diagnosis were utilised, including professional diagnosis, the Mini‐Mental State Examination (MMSE), modified questionnaires, and interviews. MMSE [28, 31, 32, 34, 35, 38, 40] and professional diagnosis [28, 29, 30, 36, 37, 39, 43, 44, 45] were the most commonly used tests, both being employed in seven out of 21 studies. Seventeen studies recruited participants who were aged over 60 years [28, 30, 31, 32, 33, 34, 35, 36, 37, 39, 40, 41, 42, 43, 45, 46, 47], while other studies included a younger population which was more than 30 years old or 40 years [29, 38, 44, 48].

### 3.2 Study Outcome and Cognition Measurement

Fourteen articles utilised dementia as the outcome variable [28, 29, 30, 32, 34, 35, 36, 37, 38, 39, 42, 43, 44, 45], while the nine articles used MCI as the outcome variable [32, 33, 36, 38, 40, 41, 46, 47, 49], Additionally, two studies examined both MCI and dementia. Diagnosis of dementia was identified through medical diagnosis in six articles [28, 29, 39, 43, 44, 45], and one employed a comprehensive cognitive test battery, including the Mini‐Mental State Examination (MMSE), Delayed Word Recall Test, Beck Depression Inventory (BDI), and Verbal Fluency Task [37].

Diagnosis of MMSE was used for mild cognitive decline assessment with one article defining < 27 as MCI [32], one article defining < 24 as MCI [34], and another one having stratified MMSE criteria according to different education levels. One research used MMI, which is a more detailed criterion of cognitive impairment including MMSE, as a diagnostic test method [33]. Other studies assessed cognitive status using various tools and methods, including the SPMSQ (Short Portable Mental Status Questionnaire) [41], the Montreal Cognitive Assessment (MoCA) [46], the Activities of Daily Living (ADL) Independence Assessment Criteria for Older Individuals with Dementia Scale [42], subjective cognitive complaints, and interviews [48].

Four studies examined both female and male populations, while only two provided OR value in both sexes [28, 35]. Seven studies focused on edentulism [28, 29, 33, 38, 40, 41, 42]. Three studies evaluated the influence of denture on cognitive function [31, 38, 39].

### 3.3 Association Between Dentition Status and Dementia

**FIGURE 2:**
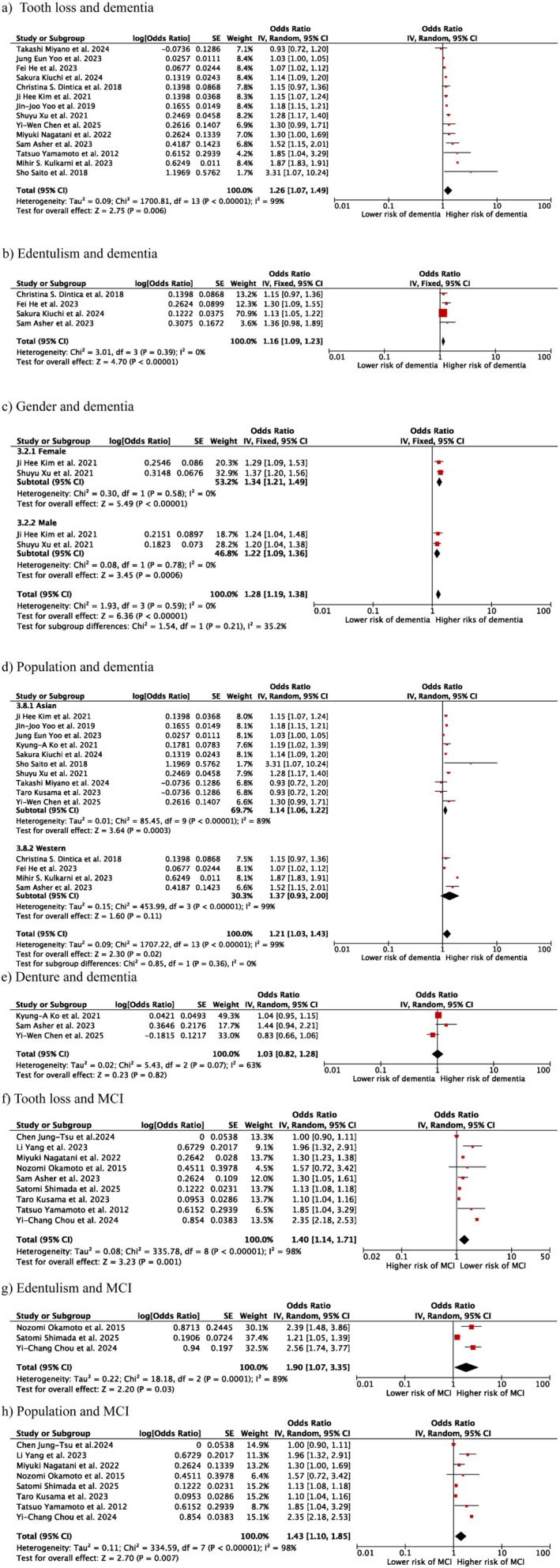
Meta‐analysis results. (a) Forest plot demonstrating the impact of tooth loss on dementia; (b) Forest plot demonstrating the impact of edentulism on dementia; (c) Forest plot demonstrating the impact of gender on dementia; (d) Forest plot demonstrating the impact of population on dementia; (e) Forest plot demonstrating the impact of denture use on dementia; (f) Forest plot demonstrating the impact of tooth loss on mild cognitive impairment; (g) Forest plot demonstrating the influence of edentulism on mild cognitive impairment; (h) Forest plot demonstrating the influence of population on mild cognitive impairment.

Thirteen of the fourteen studies demonstrated a positive correlation between tooth loss and dementia. A dose‐dependent association was observed between the severity of dentition impairment and the degree of cognitive decline, with more extensive tooth loss correlating with progressively worse dementia status [28, 29, 31, 36, 37, 50]. The pooled odds ratio among the 14 studies that examined the relationship between tooth loss and dementia was 1.26 [1.07, 1.49], with a high degree of heterogeneity (*I* ^2^ = 99%, *p* = 0.06) (Figure 2a). Four studies investigated the impact of edentulism on dementia and the pooled OR was 1.16 [1.09, 1.23] (*I* ^2^ = 0%, *p* < 0.001) (Figure 2b) [28, 29, 38, 42].

The pooled OR among men and female was also detected. A slightly elevated OR value was detected among the female group and male group, 1.22 [1.09, 1.38] and 1.34 [1.21, 1.49], respectively (Figure 2c).

The pooled OR among Asian population was 1.09 [1.08, 1.11], while it is 1.69 [1.66, 1.72] in the Western inhabitants. A substantial disparity was observed among various populations (*p* < 0.001) (Figure 2d). Three studies investigated the impact of denture on dementia; the pooled OR value was 1.03 [0.82, 1.28] (Figure 2e).

### 3.4 Association Between Dentition Status and Mild Cognitive Decline

Seven out of nine studies detected an association between tooth loss and MCI, with a pooled OR = MCI of 1.40 [1.14, 1.71] (*I* ^2^ = 89%, *p* = 0.001) (Figure 2f). The larger amount of tooth loss correlates with inferior cognitive statuses. Three studies [33, 40, 41] investigated the impact of edentulism on dementia; a pooled OR was 1.90 [1.07, 3.35] (*I* ^2^ = 89%, *p* < 0.001) (Figure 2g), higher than general tooth loss.

As there were no studies on MCI in Western populations, the pooled odds ratio was calculated only among populations from Asian countries, resulting in a value of 1.43 [1.10, 1.85] (Figure 2h).

### 3.5 Publication Bias Assessment

**FIGURE 3:**
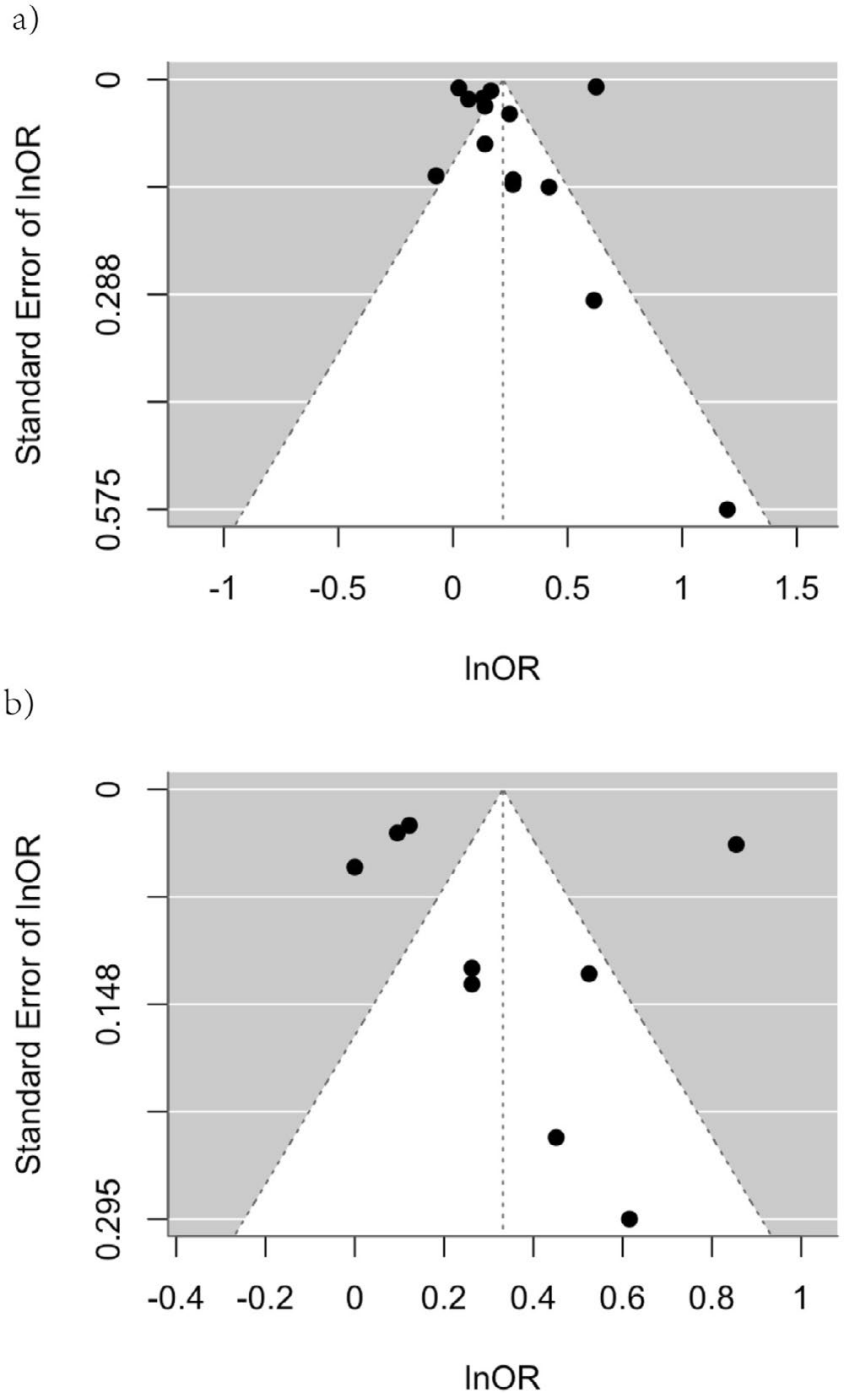
Funnel plot showing publication bias. (a) A funnel diagram that illustrates the publication bias in the studies included in the analysis of the relationship between tooth loss and dementia. (b) A funnel diagram that illustrates the publication bias in the included studies regarding the relationship between mild cognitive impairment and tooth loss.

Funnel plots were used to evaluate publication bias in this study. The funnel plot did not exhibit any significant asymmetry in the associations between tooth loss and MCI, as well as tooth loss and dementia, as determined by visual inspection. The absence of substantial publication bias was further corroborated by Egger's test, which yielded *p*‐values of 0.534 for tooth loss and dementia and 0.635 for tooth loss and MCI (Figure 3).

## 4 Discussion

Our research examined the precise correlation between cognitive decline and tooth loss in longitudinal studies that enrolled 35 744 989 participants. This research represents the most comprehensive review of longitudinal cohort studies that investigate the relationship between cognitive impairment and tooth loss. In comparison to prior systematic reviews [50, 51, 52, 53, 54], our study provides a greater statistical power to identify potential interplay between compromised dentition and cognition by integrating data from 21 original longitudinal studies with an average tracking period of 8.87 years.

The findings suggested that lost teeth increase the likelihood of dementia by 1.26 times and cognitive decline by 1.40 times.

Potential mechanisms in tooth‐loss‐cognition‐link have been explored previously. Research involving human participants has demonstrated changes in brain volume and function among individuals with tooth loss [28, 55]. Complementing these findings, laboratory studies using animal models have provided further insights. For example, rodents that lost their teeth exhibited a decrease in the number of neurons in the hippocampus [56]. Additionally, impaired synaptic function and delayed nerve growth were reported in Sprague–Dawley (SD) rats with tooth loss [57]. Research conducted on animal models has suggested that the decrease in afferent stimulation of peripheral receptors, which may be a consequence of the loss of occlusal support, may contribute to neurodegenerative symptoms [58]. The potential pathways connecting tooth loss to cognitive impairment can be summarised into three key mechanisms: (1) dysfunction of peripheral receptors, (2) neurodegenerative changes caused by nutrition deficit, and (3) oxidative stress induced by chronic inflammation [58]. Collectively, these studies suggest that tooth loss may negatively impact brain structure and function, potentially through reduced sensory input and decreased neural stimulation.

An insight into the dose‐dependent impact of tooth loss on cognitive performance can be shown in the OR ratio of the edentulous group compared to dentate people. The higher OR ratio in the edentulous group (1.16 [1.09, 1.23] for dementia and 1.90 [1.07, 3.35] for MCI) means that the severity of tooth loss has a proportionally enhanced effect on cognitive decline. The results are consistent with published cohort studies [59, 60]. The underlying reason can be largely explained by poorer mastication ability and a severe decrease in sensory stimulation from the dental cavity to the brain caused by edentulism [61].

On the other hand, tooth loss may be partially compensated by the application of prostheses. This is supported by our findings, which indicated a nonsignificant impact of tooth loss on dementia risk among denture wearers, suggesting that dentures may mitigate the negative impacts of tooth loss on cognitive performance. This protective effect of dentures is partially due to the restoration of mastication ability and neurosensory stimulation conduction [62, 63]. Previous research has demonstrated that mastication acted as a modulator of hippocampal function, promoting its functional reversibility [64]. Furthermore, animal studies have shown that enhanced chewing ability can rescue stress‐induced suppression of chronic potentiation in the hippocampus, thereby positively influencing cognitive cognition [65].

Furthermore, maintaining proper oral hygiene can mitigate the likelihood of cognitive impairments associated with missing teeth as reported in Jung's study [31]. These findings indicate that localised oral inflammation serves as a critical biological link between dentition and cognitive health, highlighting dental plaque management as a modifiable dementia prevention target. It is important to acknowledge that individuals with more robust cognitive abilities are generally more proactive in their oral hygiene practices, whereas those with weaker cognitive abilities may face challenges in independently managing their oral health [54].

In our subgroup analysis, the disparity between genders was spotted in tooth loss‐related cognitive decline with a greater risk found in the female group. This discovery is consistent with prior research that has demonstrated that the sex of the female individual is a distinct risk factor for the development and progression of dementia [66, 67]. Likewise, a higher incidence of dementia was also reported in the female subset in previous studies conducted in Europe [68, 69]. One potential explanation for this disparity may be the role of APOE, a major susceptibility gene for dementia, which appears to have a more pronounced impact on women [70, 71]. In addition to genetic factors, the longer life expectancy of women compared to men may indirectly contribute to their increased risk of both tooth loss and cognitive deficit.

Various diagnostic tools were utilised in the studies included, with the professional diagnosis being the most commonly employed tool, while others utilised modified versions of the MMSE, interviews, or scales. Although MMSE score has a high accuracy specificity in the screening of dementia, it cannot replace professional diagnosis [72, 73]. Therefore, it is critical to note that studies based on the MMSE test, as well as other diagnostic interviews or scales, for dementia identification may potentially introduce bias by not prioritising professional diagnosis. In the present review, it is important to note that the cutting‐off points used differed greatly in the diagnosis of MCI: One study chose 27 as the cutting‐off point [32], with another using 24 as the criterion [34]. Whereas previous literature reviews indicate that the MMSE cutoff score of 27 is widely adopted, as it provides an optimal balance between sensitivity and specificity [74]. Such a kind of diagnostic difference in this project might be a source of high heterogeneity in this meta‐analysis.

There are some limitations in this review and meta‐analysis. Firstly, the preservation of teeth may be influenced by cognitive function, as individuals with dementia frequently experience symptoms such as physical dysfunction and difficulty sustaining oral health. The reverse impact of cognitive function on tooth loss remains to be investigated in the future, as the current study did not concentrate on the reverse causality. Secondly, the fact that women are at a higher risk of cognitive impairment than males suggests that gender stratification should be investigated in future research. Lastly, longitudinal studies investigating confounding factors are required to elucidate the interconnections between tooth loss and cognition, as well as the potential relationship among the confounders.

## 5 Conclusion

The current review emphasises that tooth loss may serve as an important risk factor for the development of dementia and mild cognitive decline. Results elucidated the causal relationship between cognitive function and tooth loss through longitudinal studies. The risk of cognitive decline is associated with the severity of dental loss, as indicated by the studies that were included. Development of cognitive decline is more prevalent among women. Dentures can be employed to mitigate the increase in risk associated with tooth loss.

From a clinical perspective, this systematic review and meta‐analysis offer clinical evidence of the impact of tooth loss on mild cognitive impairment and dementia, emphasising the importance of dental preservation and restoration in terms of cognition. Our work provides an update of relevant research and deepens the understanding of tooth loss and cognitive impairment. The elucidation of the impact of tooth loss on cognitive decline and dementia is beneficial for the elderly population in order to achieve healthy longevity.

## Author Contributions

Huimin Chen was responsible for study design, data collection, and drafting the manuscript. Ling Ji contributed to data curation and analysis. Yilin Wang assisted with data analysis and manuscript revision. Iven Klineberg helped with manuscript revision. Hui Chen participated in data interpretation and manuscript revision. All authors have approved the final version of the manuscript.

## Funding

The authors have nothing to report.

## Ethics Statement

The authors have nothing to report.

## Conflicts of Interest

The authors declare no conflicts of interest.

## Data Availability

The data that support the findings of this study are available from the corresponding author upon reasonable request.

## References

[1] E. Nichols , J. D. Steinmetz , S. E. Vollset , et al., “Estimation of the Global Prevalence of Dementia in 2019 and Forecasted Prevalence in 2050: An Analysis for the Global Burden of Disease Study 2019,” Lancet Public Health 7, no. 2 (2022): e105–e125, 10.1016/S2468-2667(21)00249-8. 34998485 PMC8810394

[2] R. G. Morris , “Working Memory in Alzheimer‐Type Dementia,” Neuropsychology 8, no. 4 (1994): 544–554, 10.1037/0894-4105.8.4.544.

[3] B. E. Murdoch , H. J. Chenery , V. Wilks , and R. S. Boyle , “Language Disorders in Dementia of the Alzheimer Type,” Brain and Language 31, no. 1 (1987): 122–137, 10.1016/0093-934X(87)90064-2. 2437993

[4] P. Suttanon , K. D. Hill , C. M. Said , D. LoGiudice , N. T. Lautenschlager , and K. J. Dodd , “Balance and Mobility Dysfunction and Falls Risk in Older People With Mild to Moderate Alzheimer Disease,” American Journal of Physical Medicine & Rehabilitation 91, no. 1 (2012): 12–23, 10.1097/PHM.0b013e31823caeea. 22157433

[5] J. Xu , Y. Zhang , C. Qiu , and F. Cheng , “Global and Regional Economic Costs of Dementia: A Systematic Review,” Lancet 390 (2017): S47, 10.1016/S0140-6736(17)33185-9.

[6] S. Banerjee , “Quality of Life in Dementia: More Than Just Cognition. An Analysis of Associations With Quality of Life in Dementia,” Journal of Neurology, Neurosurgery & Psychiatry 77, no. 2 (2006): 146–148, 10.1136/jnnp.2005.072983. 16421113 PMC2077592

[7] S. Gauthier , B. Reisberg , M. Zaudig , et al., “Mild Cognitive Impairment,” Lancet 367, no. 9518 (2006): 1262–1270, 10.1016/S0140-6736(06)68542-5. 16631882

[8] K. Schmidtke and S. Hermeneit , “High Rate of Conversion to Alzheimer's Disease in a Cohort of Amnestic MCI Patients,” International Psychogeriatrics 20, no. 1 (2008): 96–108, 10.1017/S1041610207005509. 17506911

[9] J. H. Chen , K. P. Lin , and Y. C. Chen , “Risk Factors for Dementia,” Journal of the Formosan Medical Association 108, no. 10 (2009): 754–764, 10.1016/S0929-6646(09)60402-2. 19864195

[10] L. Li , Q. Zhang , D. Yang , et al., “Tooth Loss and the Risk of Cognitive Decline and Dementia: A Meta‐Analysis of Cohort Studies,” Frontiers in Neurology 14 (2023): 1103052, 10.3389/fneur.2023.1103052. 37139053 PMC10150074

[11] J. Chen , C. J. Ren , L. Wu , et al., “Tooth Loss Is Associated With Increased Risk of Dementia and With a Dose‐Response Relationship,” Frontiers in Aging Neuroscience 10 (2018): 415, 10.3389/fnagi.2018.00415. 30618721 PMC6305430

[12] World Health Organization , “Ageing and Health,” (2022), https://www.who.int/news‐room/fact‐sheets/detail/ageing‐and‐health.

[13] A. E. Gerritsen , P. F. Allen , D. J. Witter , E. M. Bronkhorst , and N. H. J. Creugers , “Tooth Loss and Oral Health‐Related Quality of Life: A Systematic Review and Meta‐Analysis,” Health and Quality of Life Outcomes 8 (2010): 126, 10.1186/1477-7525-8-126. 21050499 PMC2992503

[14] E. B. Özhayat , “Influence of Self‐Esteem and Negative Affectivity on Oral Health‐Related Quality of Life in Patients With Partial Tooth Loss,” Community Dentistry and Oral Epidemiology (2012), 10.1111/cdoe.12032. 23253094

[15] H. C. Hung , G. Colditz , and K. J. Joshipura , “The Association Between Tooth Loss and the Self‐Reported Intake of Selected CVD‐Related Nutrients and Foods Among US Women,” Community Dentistry and Oral Epidemiology 33, no. 3 (2005): 167–173, 10.1111/j.1600-0528.2005.00200.x. 15853839

[16] C. A. Geissler and J. F. Bates , “The Nutritional Effects of Tooth Loss,” American Journal of Clinical Nutrition 39, no. 3 (1984): 478–489, 10.1093/ajcn/39.3.478. 6364777

[17] P. Galindo‐Moreno , L. Lopez‐Chaichio , M. Padial‐Molina , et al., “The Impact of Tooth Loss on Cognitive Function,” Clinical Oral Investigations 26, no. 4 (2022): 3493–3500, 10.1007/s00784-021-04318-4. 34881401 PMC8979879

[18] F. Shinsho , “New Strategy for Better Geriatric Oral Health in Japan: 80/20 Movement and Healthy Japan 21,” International Dental Journal 51 (2001): 200–206, 10.1002/j.1875-595X.2001.tb00867.x. 11561879

[19] K. y. Kubo , A. Ogasawara , H. Tsugane , M. Iinuma , T. Takahashi , and K. Azuma , “Environmental Enrichment Improves Hypomyelination, Synaptic Alterations, and Memory Deficits Caused by Tooth Loss in Aged SAMP8 Mice,” Archives of Oral Biology 123 (2021): 105039, 10.1016/j.archoralbio.2021.105039. 33454419

[20] T. Kobayashi , M. Kubota , T. Takahashi , et al., “Effects of Tooth Loss on Brain Structure: A Voxel‐Based Morphometry Study,” Journal of Prosthodontic Research 62, no. 3 (2018): 337–341, 10.1016/j.jpor.2017.12.007. 29475808

[21] R. T. F. Costa , J. P. J. De Oliveira Limirio , B. C. D. E. Vasconcelos , E. P. Pellizzer , and S. L. D. D. Moraes , “Rehabilitation With Dental Prostheses and Its Influence on Brain Activity: A Systematic Review,” Journal of Prosthetic Dentistry (2022): S0022391322000907, 10.1016/j.prosdent.2022.02.007. 35305835

[22] D. Cerutti‐Kopplin , J. Feine , D. M. Padilha , et al., “Tooth Loss Increases the Risk of Diminished Cognitive Function: A Systematic Review and Meta‐Analysis,” JDR Clinical & Translational Research 1, no. 1 (2016): 10–19, 10.1177/2380084416633102. 30931697

[23] PRISMA‐P Group , D. Moher , L. Shamseer , et al., “Preferred Reporting Items for Systematic Review and Meta‐Analysis Protocols (PRISMA‐P) 2015 Statement,” Systematic Reviews 4, no. 1 (2015): 1, 10.1186/2046-4053-4-1. 25554246 PMC4320440

[24] G. A. Wells , B. Shea , D. O'Connell , et al., The Newcastle‐Ottawa Scale (NOS) for Assessing the Quality of Nonrandomised Studies in Meta‐Analyses (Ottawa Hospital Research Institute, 2011), accessed September 5, 2021, https://ohri.ca/en/who‐we‐are/core‐facilities‐and‐platforms/ottawa‐methods‐centre/newcastle‐ottawa‐scale.

[25] A. J. Viera , “Odds Ratios and Risk Ratios: What's the Difference and Why Does It Matter?,” Southern Medical Journal 101, no. 7 (2008): 730–734, 10.1097/SMJ.0b013e31817a7ee4. 18580722

[26] S. Chinn , “A Simple Method for Converting an Odds Ratio to Effect Size for Use in Meta‐Analysis,” Statistics in Medicine 19, no. 22 (2000): 3127–3131, 10.1002/1097-0258(20001130)19:22<>3.0.CO;2-M. 11113947

[27] M. Egger , G. D. Smith , and A. N. Phillips , “Meta‐Analysis: Principles and Procedures,” BMJ 315, no. 7121 (1997): 1533–1537, 10.1136/bmj.315.7121.1533. 9432252 PMC2127925

[28] C. S. Dintica , D. Rizzuto , A. Marseglia , et al., “Tooth Loss Is Associated With Accelerated Cognitive Decline and Volumetric Brain Differences: A Population‐Based Study,” Neurobiology of Aging 67 (2018): 23–30, 10.1016/j.neurobiolaging.2018.03.003. 29609079

[29] F. He , H. Luo , L. Yin , et al., “Poor Oral Health as a Risk Factor for Dementia in a Swedish Population: A Cohort Study With 40 Years of Follow‐Up,” Journal of Alzheimer's Disease 92, no. 1 (2023): 171–181, 10.3233/JAD-215177. 36710668

[30] J. H. Kim , J. K. Oh , J. H. Wee , Y. H. Kim , S. H. Byun , and H. G. Choi , “Association Between Tooth Loss and Alzheimer's Disease in a Nested Case–Control Study Based on a National Health Screening Cohort,” Journal of Clinical Medicine 10, no. 17 (2021): 3763, 10.3390/jcm10173763. 34501210 PMC8432055

[31] K. A. Ko , J. Y. Park , J. S. Lee , et al., “The Impact of Masticatory Function on Cognitive Impairment in Older Patients: A Population‐Based Matched Case‐Control Study,” Yonsei Medical Journal 63, no. 8 (2022): 783–789, 10.3349/ymj.2022.63.8.783. 35914761 PMC9344271

[32] M. Nagatani , T. Tanaka , B. K. Son , et al., “Oral Frailty as a Risk Factor for Mild Cognitive Impairment in Community‐Dwelling Older Adults: Kashiwa Study,” Experimental Gerontology 172 (2023): 112075, 10.1016/j.exger.2022.112075. 36581224

[33] N. Okamoto , M. Morikawa , K. Tomioka , M. Yanagi , N. Amano , and N. Kurumatani , “Association Between Tooth Loss and the Development of Mild Memory Impairment in the Elderly: The Fujiwara‐Kyo Study,” Journal of Alzheimer's Disease 44, no. 3 (2015): 777–786, 10.3233/JAD-141665. 25362033

[34] S. Saito , T. Ohi , T. Murakami , et al., “Association Between Tooth Loss and Cognitive Impairment in Community‐Dwelling Older Japanese Adults: A 4‐Year Prospective Cohort Study From the Ohasama Study,” BMC Oral Health 18, no. 1 (2018): 142, 10.1186/s12903-018-0602-7. 30126407 PMC6102919

[35] S. Xu , X. Huang , Y. Gong , and J. Sun , “Association Between Tooth Loss Rate and Risk of Mild Cognitive Impairment in Older Adults: A Population‐Based Longitudinal Study,” Aging 13, no. 17 (2021): 21599–21609, 10.18632/aging.203504. 34495870 PMC8457613

[36] T. Yamamoto , K. Kondo , H. Hirai , M. Nakade , J. Aida , and Y. Hirata , “Association Between Self‐Reported Dental Health Status and Onset of Dementia: A 4‐Year Prospective Cohort Study of Older Japanese Adults From the Aichi Gerontological Evaluation Study (AGES) Project,” Psychosomatic Medicine 74, no. 3 (2012): 241–248, 10.1097/PSY.0b013e318246dffb. 22408130

[37] J. J. Yoo , J. H. Yoon , M. J. Kang , M. Kim , and N. Oh , “The Effect of Missing Teeth on Dementia in Older People: A Nationwide Population‐Based Cohort Study in South Korea,” BMC Oral Health 19, no. 1 (2019): 61, 10.1186/s12903-019-0750-4. 31023356 PMC6485168

[38] S. Asher , A. L. Suominen , R. Stephen , T. Ngandu , S. Koskinen , and A. Solomon , “Association of Tooth Count With Cognitive Decline and Dementia in the Finnish Adult Population,” Journal of Clinical Periodontology 50, no. 9 (2023): 1154–1166, 10.1111/jcpe.13851. 37461219

[39] Y. W. Chen , C. Y. Li , S. C. Lee , et al., “Associations Among Tooth Loss, Denture Use, and Dementia in Older Taiwanese Adults,” Journal of the Chinese Medical Association (2024), 10.1097/JCMA.0000000000001171. PMC12718786 39774060

[40] S. Shimada , Y. Matsuyama , and J. Aida , “Tooth Loss Explains Income Inequalities in Dementia,” Journal of Dentistry 153 (2025): 105518, 10.1016/j.jdent.2024.105518. 39653269

[41] Y. C. Chou , S. H. Weng , F. S. Cheng , and H. Y. Hu , “Denture Use Mitigates the Cognitive Impact of Tooth Loss in Older Adults,” Journals of Gerontology. Series A, Biological Sciences and Medical Sciences 80, no. 1 (2024): glae248, 10.1093/gerona/glae248. 39626020

[42] S. Kiuchi , Y. Matsuyama , K. Takeuchi , et al., “Number of Teeth and Dementia‐Free Life Expectancy: A 10‐Year Follow‐Up Study From the Japan Gerontological Evaluation Study,” Journal of the American Medical Directors Association 25, no. 11 (2024): 105258, 10.1016/j.jamda.2024.105258. 39276797

[43] T. Miyano , Y. Ayukawa , T. Anada , et al., “Association Between Reduced Posterior Occlusal Contact and Alzheimer's Disease Onset in Older Japanese Adults: Results From the LIFE Study,” Journal of Alzheimer's Disease 97, no. 2 (2024): 871–881, 10.3233/JAD-230449. PMC10894584 38160352

[44] J. E. Yoo , Y. Huh , S. H. Park , et al., “Association Between Dental Diseases and Oral Hygiene Care and the Risk of Dementia: A Retrospective Cohort Study,” Journal of the American Medical Directors Association 24, no. 12 (2023): 1924–1930.e3, 10.1016/j.jamda.2023.08.011. 37709259

[45] M. S. Kulkarni , B. C. Miller , M. Mahani , et al., “Poor Oral Health Linked With Higher Risk of Alzheimer's Disease,” Brain Sciences 13, no. 11 (2023): 1555, 10.3390/brainsci13111555. 38002515 PMC10669972

[46] J. T. Chen , S. Tsai , M. H. Chen , et al., “Association Between Oral Health and Cognitive Impairment in Older Adults: Insights From a Six‐Year Prospective Cohort Study,” Journal of Dentistry 147 (2024): 105088, 10.1016/j.jdent.2024.105088. 38801941

[47] T. Kusama , K. Takeuchi , S. Kiuchi , J. Aida , and K. Osaka , “Poor Oral Health and Dementia Risk Under Time‐Varying Confounding: A Cohort Study Based on Marginal Structural Models,” Journal of the American Geriatrics Society 72, no. 3 (2024): 729–741, 10.1111/jgs.18707. 38064294

[48] Y. Li , C. L. Huang , X. Z. Lu , et al., “Longitudinal Association of Edentulism With Cognitive Impairment, Sarcopenia and All‐Cause Mortality Among Older Chinese Adults,” BMC Oral Health 23, no. 1 (2023): 333, 10.1186/s12903-023-03015-w. 37244990 PMC10225090

[49] H. L. Yang , F. R. Li , P. L. Chen , X. Cheng , C. Mao , and X. B. Wu , “Tooth Loss, Denture Use, and Cognitive Impairment in Chinese Older Adults: A Community Cohort Study,” Journals of Gerontology: Series A 77, no. 1 (2022): 180–187, 10.1093/gerona/glab056. 33674815

[50] X. Qi , Z. Zhu , B. L. Plassman , and B. Wu , “Dose‐Response Meta‐Analysis on Tooth Loss With the Risk of Cognitive Impairment and Dementia,” Journal of the American Medical Directors Association 22, no. 10 (2021): 2039–2045, 10.1016/j.jamda.2021.05.009. 34579934 PMC8479246

[51] S. Asher , R. Stephen , P. Mäntylä , A. L. Suominen , and A. Solomon , “Periodontal Health, Cognitive Decline, and Dementia: A Systematic Review and Meta‐Analysis of Longitudinal Studies,” Journal of the American Geriatrics Society 70, no. 9 (2022): 2695–2709, 10.1111/jgs.17978. 36073186 PMC9826143

[52] W. l. Fang , M. j. Jiang , B. b. Gu , et al., “Tooth Loss as a Risk Factor for Dementia: Systematic Review and Meta‐Analysis of 21 Observational Studies,” BMC Psychiatry 18, no. 1 (2018): 345, 10.1186/s12888-018-1927-0. 30342524 PMC6195976

[53] B. Agarwal , M. E. Bizzoca , G. Musella , et al., “Tooth Loss in Periodontitis Patients—A Risk Factor for Mild Cognitive Impairment: A Systematic Review and Meta—Analysis,” Journal of Personalized Medicine 14, no. 9 (2024): 953, 10.3390/jpm14090953. 39338207 PMC11433130

[54] M. Dioguardi , G. Di Gioia , G. A. Caloro , et al., “The Association Between Tooth Loss and Alzheimer's Disease: A Systematic Review With Meta‐Analysis of Case Control Studies,” Dentistry Journal 7, no. 2 (2019): 49, 10.3390/dj7020049. 31052367 PMC6630622

[55] Y. Matsuyama , T. Fujiwara , H. Murayama , M. Machida , S. Inoue , and Y. Shobugawa , “Differences in Brain Volume by Tooth Loss and Cognitive Function in Older Japanese Adults,” American Journal of Geriatric Psychiatry 30, no. 12 (2022): 1271–1279, 10.1016/j.jagp.2022.06.005. 35831211

[56] H. Oue , Y. Miyamoto , S. Okada , et al., “Tooth Loss Induces Memory Impairment and Neuronal Cell Loss in APP Transgenic Mice,” Behavioural Brain Research 252 (2013): 318–325, 10.1016/j.bbr.2013.06.015. 23773908

[57] K. Kubota , K. Nagae , S. Shibanai , et al., “Degenerative Changes of Primary Neurons Following Tooth Extraction,” Anatomischer Anzeiger 166, no. 1–5 (1988): 133–139. 3263820

[58] X. Wang , J. Hu , and Q. Jiang , “Tooth Loss‐Associated Mechanisms That Negatively Affect Cognitive Function: A Systematic Review of Animal Experiments Based on Occlusal Support Loss and Cognitive Impairment,” Frontiers in Neuroscience 16 (2022): 811335, 10.3389/fnins.2022.811335. 35221901 PMC8866659

[59] B. Wu , H. Luo , C. Tan , et al., “Diabetes, Edentulism, and Cognitive Decline: A 12‐Year Prospective Analysis,” Journal of Dental Research 102 (2023): 002203452311558, 10.1177/00220345231155825. PMC10399080 36908186

[60] J. A. Jones , K. Moss , T. L. Finlayson , J. S. Preisser , and J. A. Weintraub , “Edentulism Predicts Cognitive Decline in the US Health and Retirement Cohort Study,” Journal of Dental Research 102 (2023): 00220345231167805, 10.1177/00220345231167805. PMC10399082 37314011

[61] A. Tada and H. Miura , “Association Between Mastication and Cognitive Status: A Systematic Review,” Archives of Gerontology and Geriatrics 70 (2017): 44–53, 10.1016/j.archger.2016.12.006. 28042986

[62] D. Cerutti‐Kopplin , E. Emami , J. B. Hilgert , F. N. Hugo , and D. M. P. Padilha , “Cognitive Status of Edentate Elders Wearing Complete Denture: Does Quality of Denture Matter?,” Journal of Dentistry 43, no. 9 (2015): 1071–1075, 10.1016/j.jdent.2015.07.008. 26188327

[63] M. S. Kim , B. Oh , J. W. Yoo , and D. H. Han , “The Association Between Mastication and Mild Cognitive Impairment in Korean Adults,” Medicine 99, no. 23 (2020): e20653, 10.1097/MD.0000000000020653. 32502052 PMC7306381

[64] Y. Ono , T. Yamamoto , K. Y. Kubo , and M. Onozuka , “Occlusion and Brain Function: Mastication as a Prevention of Cognitive Dysfunction,” Journal of Oral Rehabilitation (2010), 10.1111/j.1365-2842.2010.02079.x. 20236235

[65] K. Watanabe , S. Ozono , K. Nishiyama , et al., “The Molarless Condition in Aged SAMP8 Mice Attenuates Hippocampal Fos Induction Linked to Water Maze Performance,” Behavioural Brain Research 128, no. 1 (2002): 19–25, 10.1016/S0166-4328(01)00268-6. 11755686

[66] S. Artero , M. L. Ancelin , F. Portet , et al., “Risk Profiles for Mild Cognitive Impairment and Progression to Dementia Are Gender Specific,” Journal of Neurology, Neurosurgery & Psychiatry 79, no. 9 (2008): 979–984, 10.1136/jnnp.2007.136903. 18450788

[67] M. Zhang , R. Katzman , D. Salmon , et al., “The Prevalence of Dementia and Alzheimer's Disease in Shanghai, China: Impact of Age, Gender, and Education,” Annals of Neurology 27, no. 4 (1990): 428–437, 10.1002/ana.410270412. 2353798

[68] A. Hofman , W. A. Rocca , C. Brayne , et al., “The Prevalence of Dementia in Europe: A Collaborative Study of 1980–1990 Findings,” International Journal of Epidemiology 20, no. 3 (1991): 736–748, 10.1093/ije/20.3.736. 1955260

[69] K. Andersen , L. J. Launer , M. E. Dewey , et al., “Gender Differences in the Incidence of AD and Vascular Dementia: The EURODEM Studies,” Neurology 53, no. 9 (1999): 1992, 10.1212/WNL.53.9.1992. 10599770

[70] E. H. Corder , A. M. Saunders , W. J. Strittmatter , et al., “Gene Dose of Apolipoprotein E Type 4 Allele and the Risk of Alzheimer's Disease in Late Onset Families,” Science 261, no. 5123 (1993): 921–923, 10.1126/science.8346443. 8346443

[71] E. Ghebremedhin , C. Schultz , D. R. Thal , et al., “Gender and Age Modify the Association Between APOE and AD‐Related Neuropathology,” Neurology 56, no. 12 (2001): 1696–1701, 10.1212/WNL.56.12.1696. 11425936

[72] W. A. Kukull , E. B. Larson , L. Teri , J. Bowen , W. McCormick , and M. L. Pfanschmidt , “The Mini‐Mental State Examination Score and the Clinical Diagnosis of Dementia,” Journal of Clinical Epidemiology 47, no. 9 (1994): 1061–1067, 10.1016/0895-4356(94)90122-8. 7730909

[73] A. J. Mitchell , “A Meta‐Analysis of the Accuracy of the Mini‐Mental State Examination in the Detection of Dementia and Mild Cognitive Impairment,” Journal of Psychiatric Research 43, no. 4 (2009): 411–431, 10.1016/j.jpsychires.2008.04.014. 18579155

[74] C. T. Chun , K. Seward , A. Patterson , A. Melton , and L. MacDonald‐Wicks , “Evaluation of Available Cognitive Tools Used to Measure Mild Cognitive Decline: A Scoping Review,” Nutrients 13, no. 11 (2021): 3974, 10.3390/nu13113974. 34836228 PMC8623828

